# Medical teachers’ experience of emergency remote teaching during the COVID-19 pandemic: a cross-institutional study

**DOI:** 10.1186/s12909-022-03367-x

**Published:** 2022-04-21

**Authors:** Enoch Chan, Mei Li Khong, Adrienne Torda, Julian A. Tanner, Gary M. Velan, Gordon T. C. Wong

**Affiliations:** 1 School of Clinical Medicine, The University of Hong Kong, Pokfulam, Hong Kong SAR, China; 2 Office of Medical Education, Faculty of Medicine & Health, The University of New South Wales, Sydney, NSW 2052 Australia; 3 School of Biomedical Sciences, The University of Hong Kong, Pokfulam, Hong Kong SAR, China; 4 School of Medical Sciences, Faculty of Medicine & Health, The University of New South Wales, Sydney, NSW 2052 Australia; 5 Department of Anaesthesiology, School of Clinical Medicine, The University of Hong Kong, Pokfulam, Hong Kong SAR, China

**Keywords:** COVID-19, Medical education, Healthcare education, Online teaching, Pandemic, Remote teaching

## Abstract

**Background:**

The COVID-19 pandemic and the consequent social distancing measures caused unprecedented disruption for medical and healthcare education. This study examined medical teachers’ experience with emergency remote teaching during the pandemic and their acceptance of online teaching after the pandemic.

**Methods:**

In this sequential mixed methods study, online surveys were disseminated to teachers (n = 139) at two Asia–Pacific medical schools to evaluate their experience with emergency remote teaching during the pandemic. Subsequently, in-depth interviews were conducted with teachers from both institutions (n = 13). Each interviewee was classified into an adopter category based on Rogers’ Diffusion of Innovations Theory. Interview transcripts were analyzed thematically, and the descriptive themes were mapped to broader themes partly based on the Technology Acceptance Model and these included: (i) perceived usefulness of online teaching, (ii) perceived ease of delivering online teaching, (iii) experience with institutional support and (iv) acceptance of online teaching after the pandemic.

**Results:**

Our participants described accounts of successes with their emergency remote teaching and difficulties they experienced. In general, most participants found it difficult to deliver clinical skills teaching remotely and manage large groups of students in synchronous online classes. With regards to institutional support, teachers with lower technological literacy required just-in-time technical support, while teachers who were innovative in their online teaching practices found that IT support alone could not fully address their needs. It was also found that teachers’ acceptance of online teaching after the pandemic was influenced by their belief about the usefulness of online teaching.

**Conclusions:**

This study demonstrated that our participants managed to adapt to emergency remote teaching during this pandemic, and it also identified a myriad of drivers and blockers to online teaching adoption for medical teachers. It highlights the need for institutes to better support their teaching staff with diverse needs in their online teaching.

**Supplementary Information:**

The online version contains supplementary material available at 10.1186/s12909-022-03367-x.

## Introduction

The global pandemic of coronavirus disease 2019 (COVID-19) has disrupted students’ access to physical campuses worldwide for the majority of year 2020 [1]. Stringent infection control measures within teaching hospitals also prevented medical students’ access to clinical areas [2]. The situation posed significant challenges to the delivery of medical and healthcare education, particularly for practical elements such as clinical skills, patient encounters and laboratory sessions that had traditionally been taught face-to-face.

Prior to the pandemic, pre-recorded lecture videos, real-time lecture capturing, adaptive learning modules or online quizzes in learning management systems had been used to supplement or enhance face-to-face teaching for non-distance degree programs [3–5], and this “blended learning” approach had been regarded as the “new normal” in the higher education sector before the pandemic [6–8]. The adoption of blended teaching in the higher education setting had been previously studied using Rogers’ Diffusion of Innovations (DoI) theory [9–12]. According to this theory, innovations or technology are “diffused” (i.e. gradually communicated and adopted) among the members of a population overtime. When an innovation is introduced, it would first be adopted by a relatively small number of individuals (“innovators” and “early adopters”) and then the majority of the population (“early majority” and “late majority”) would gradually follow. However, a relatively small number of individuals (“laggards”) would remain resistant to that innovation even after an extended period since its introduction [13]. Table 1 outlines the common traits of different innovation adopters. Studies have found that teachers’ adoption of online teaching in higher education settings can be influenced by factors such as advocacy by senior management, infrastructure, equipment availability, time allowed for preparation and technological literacy [9, 10, 14–17].

**Table 1:** Characteristics of Rogers’ five categories of innovation adopters. Adopted from Humbert [9], Porter and Graham [10], Porter, Graham, Spring and Welch [12], Rogers [13]

Category	Characteristics
Innovators	The first 2.5% of individuals to adopt an innovation / technology They have substantial technical expertise and self-learn new technologies They are always ready to adopt a new technology for their day-to-day work
Early adopters	The 13.5% of individuals who adopt after the innovators They have good technical expertise and explore new technologies They adopt new technologies with more discretion than innovators They serve as examples and opinion leaders among other potential adopters
Early majority	The 34% of individuals who adopt after the early adopters They are fairly comfortable with technology They need incentives, compelling evidence of efficiency and recommendation from opinion leaders to adopt new innovations
Late majority	The 34% of individuals who adopt after the early majority They are less comfortable with technology compared to the early majority They only adopt a new technology under peer pressure or necessity They require suitable technical support for adopt new innovations
Laggards	The last 16% of individuals who adopt an innovation (if ever) They resist adopting to new innovations even under peer pressure or necessity They tend to be disconnected from the social network of their system

On the other hand, the Technology Acceptance Model (TAM) outlines the factors that determine an individual’s acceptance of technology [18]. It suggests that perceived usefulness and perceived ease of use work in concert to influence an individual’s attitude towards a technology or innovation, which then gives rise to the behavioral outcome of using or not using the technology [19]. Perceived usefulness is defined as the degree to which a person believes that using such technology or innovation would enhance their performance of a task [18], while perceived ease of use is the degree to which a person believes that using a particular technology or innovation would be free of effort. TAM has been a widely adopted model for understanding user acceptance of information technology in educational settings. A meta-analysis of 63 studies confirmed that TAM is a powerful tool for explaining teachers’ adoption of digital technology for educational purposes in general [20]. A recent study on teachers’ technology adoption drew a connection between the DoI theory and the TAM. It was found that perceived usefulness and perceived ease of use were significantly higher among highly innovative teachers in comparison to less innovative teachers, suggesting the influence of personal innovativeness on technology acceptance of teachers [21].

The mandatory adoption of online teaching (or “emergency remote teaching”) during the COVID-19 pandemic was substantially different from the introduction of blended or online teaching before the pandemic, which would have followed the pattern described by the DoI theory. During the pandemic, nearly all teachers were compelled to adopt online teaching immediately, and the time allowed for their adjustment was substantially shorter than in a non-emergency or voluntary setting [22]. It was also argued that emergency remote teaching is not the same as blended learning, as the former would have lacked a well-thought-out step-by-step process in their design, unlike the latter [23, 24]. Moreover, teaching activities such as small-group tutorials, practical classes, clinical skills demonstration and bedside teaching were mostly delivered face-to-face before the pandemic, especially for non-distance medical and healthcare education programs. However, teachers had little choice but to adapt these teaching activities to online delivery when face-to-face teaching was prohibited by infection control measures [25–29].

### Purpose

This study aimed at characterizing teachers’ experience of emergency remote teaching for undergraduate medicine and healthcare students during the COVID-19 pandemic. Broadly, this study aimed to answer the following research questions:

- How effective were their emergency remote teaching during the pandemic?
- What were the difficulties faced by teachers in their emergency remote teaching?
- How did they feel about the institutional support they received?
- What are their opinions and perspectives on blended or online teaching after the pandemic?

## Methods

### Setting

This study was conducted at two higher education institutes, namely The University of Hong Kong (HKU) and The University of New South Wales (UNSW), Sydney, Australia. At the time of this study, Li Ka Shing Faculty of Medicine at HKU (HKUMed) employed approximately 400 full-time teaching staff and more than 2900 undergraduate students across multiple programs including Biomedical Science, Chinese Medicine, Global Health and Development, Medicine, Nursing, Pharmacy and Science (Biochemistry major). The outbreak of COVID-19 in Hong Kong in late January 2020 resulted in the suspension of all face-to-face teaching in the campus and teaching hospitals between January and September 2020. HKUMed experienced disruption to face-to-face teaching prior to the pandemic due to Hong Kong’s social unrests throughout 2019, which led to the suspension of all face-to-face teaching across all universities and schools in November 2019 [25].

In contrast, at the time of this study, the Faculty of Medicine & Health at UNSW (UNSWMH) had approximately 800 full-time academic staff, and it served more than 2200 undergraduate students across multiple programs including Medicine, Exercise Physiology, International Public Health, Medical Science and Science. The COVID-19 outbreak in Australia commencing in late February 2020 led to a transition of all teaching activity to fully online delivery around late March 2020 [30]. UNSWMH had not experienced any prolonged disruption to face-to-face teaching for the last decade prior to the COVID-19 pandemic.

### Study design

We employed a sequential mixed method study design which included an online survey followed by in-depth interviews [31]. The online survey aimed at evaluating teachers’ experience with emergency remote teaching during the COVID-19 pandemic. After analysis of the survey data, the findings were used to develop questions for in-depth interviews. The qualitative data from the in-depth interviews were thematically analyzed to help explain the quantitative results and to develop further insights.

### Researcher reflexivity

Researchers EC and MLK were teaching support staff within their institute, while AT, JAT, GMV and GTCW were senior leaders for teaching and learning within their respective institutes. All researchers were actively involved in driving blended learning initiatives within their respective institutes. This study was conceptualized by GTCW and EC. During the conceptualization, GTCW and EC assumed that a proportion of teachers would have zero experience with delivering online teaching before the COVID-19 pandemic, based on their day-to-day interactions with colleagues and inference from the DoI Theory. GTCW and EC speculated that these teachers’ resistance to online teaching before the COVID-19 pandemic was due to their unfamiliarity with online teaching technology and general resistance to changes to the status quo. GTCW and EC also assumed that all teachers would have been forced to deliver emergency remote teaching during the pandemic, and this experience may influence some teachers’ attitude to online teaching in general. The study design was presented to MLK, JAT, AT and GMV, who then contributed ideas to refine the study design. The primary researcher (EC) disseminated the online surveys, anonymized all survey data and conducted the in-depth interviews. EC and MLK conducted the qualitative data analysis independently and then combined their findings.

### Survey

The survey (Appendix 1) was constructed by EC and GTCW in a meeting, during which the research questions and the researchers’ underlying assumptions were identified, and then the survey items were constructed. The survey was then reviewed and refined by MLK and JAT. The survey featured an information statement covering the aim of the survey and a request for informed consent. Subsequently, there were close-ended questions and multiple-choice questions to assess participants’ experience with online teaching before and during the COVID-19 pandemic. Afterwards, single-item scales were employed to assess participants’ sense of readiness and technical capability in online teaching during the pandemic, their satisfaction with institutional support during the pandemic and their willingness to adopt online teaching after the pandemic. An open-text field was added at the end of the survey to collect additional comments from the participants. The online survey was created using Qualtrics (Qualtrics, Provo, UT, USA) and disseminated to all teachers via internal mass emails in May 2020 at HKUMed, and in September 2020 at UNSWMH.

A total of 139 completed surveys were collected for this study. From the HKUMed cohort, 109 completed surveys were received (response rate < 27.25%). Among these survey respondents, 47.7% (52 out of 109) were clinical academic staff, while 52.3% (57 out of 109) were non-clinical academic staff. For the UNSWMH cohort, 30 completed surveys were received (response rate < 3.75%), of which 33.3% (10 out of 30) of the respondents were clinical academic staff and 66.7% (20 out of 30) were non-clinical academic staff.

Descriptive statistics of the survey results were generated in Qualtrics. Bivariate correlations between single-item scales were determined by calculating Spearman’s rank correlation coefficient (*r*_s_) followed by two-tailed statistical significance test using IBM SPSS Statistics for Windows, version 26 (IBM Corp., Armonk, N.Y., USA). Additional comments given by each participant were manually coded. The descriptive themes identified were as followed:

- Difficult to engage students during online lessons
- Difficult to teach hands-on practical skills
- Feel self-sufficient with technology
- Need better guidance, infrastructure, support and training
- Online teaching is more interactive
- Online teaching creates additional workload / is more time consuming
- Online teaching can sustain student learning when face-to-face class is suspended.
- Prefer face-to-face teaching to online teaching
- Struggling with technology

### In-depth interview and thematic analysis

Based on the quantitative findings and descriptive themes, interview questions were constructed by EC. The interview questions were then reviewed and refined by GTCW, MLK and JAT. Individual in-depth interviews were conducted face-to-face or via video call (Zoom or Microsoft Teams) between May 2020 and October 2020. The interviews were conducted in a semi-structured manner, where the interview questions (Appendix 2) were used as prompts, while the participants were encouraged to freely talk about their experience and views. Prior to the interview, each participant was given an information statement covering the aim of the interview, followed by a brief verbal explanation by the interviewer. Afterwards, written consent was sought from each participant. The total number of teachers interviewed was 13, including 6 clinical teachers and 7 non-clinical teachers, with 8 of the participants from HKUMed, and 5 from UNSWMH (Table 2). During the in-depth interviews, participants were invited to describe their own experience with online teaching before and during the pandemic, and their attitude towards online teaching. Based on these findings, we classified each interviewee into an innovation adopter category according to the DoI theory [9–11, 13]. All interviews were audio-recorded and transcribed.

**Table 2:** Summary of the background information, pre-pandemic experience with online teaching and innovation adopter category of in-depth interview participants

Interviewee	Discipline	Modalities of online teaching	Institution	Innovation adopter category
A. Clinical teachers				
CT1	Clinical Medicine	tutorial, informal consultation, clinical skills demonstration	HKUMed	Early majority
CT2	Clinical Medicine	tutorial, clinical skills demonstration	HKUMed	Early adopter
CT3	Clinical Medicine	lecture, tutorial, clinical skills demonstration	HKUMed	Innovator
CT4	Clinical Medicine	lecture, tutorial	UNSWMH	Late majority
CT5	Exercise Physiology	lecture, tutorial, laboratory practicals	UNSWMH	Early adopter
CT6	Clinical Medicine	lecture, tutorial, clinical skills demonstration	UNSWMH	Innovator
B. Non-clinical teachers				
NT1	Biomedical Science	lecture, tutorial, laboratory demonstration	HKUMed	Late majority
NT2	Medical Humanities	lecture, tutorial, informal consultation	HKUMed	Late majority
NT3	Biomedical Science	lecture, tutorial, informal consultation	HKUMed	Innovator
NT4	Public Health	lecture, tutorial	HKUMed	Early majority
NT5	Biomedical Science	tutorial	HKUMed	Early adopter
NT6	Public Health	lecture, tutorial	UNSWMH	Laggard
NT7	Biomedical Science	lecture, tutorial, laboratory demonstration	UNSWMH	Innovator

EC conducted all the in-depth interviews and transcribed the audio recordings. All personal identifiers were then removed from the transcripts before sharing with other researchers. Each participant was asked to review and approve the transcripts from their respective in-depth interviews. Two researchers (EC and MLK) independently read and thematically analyzed all transcripts in their entirety. Thematic analysis was performed by descriptive coding using MAXQDA 2020 (VERBI Software, Berlin, Germany). The descriptive codes were then consolidated into themes. Afterwards, EC and MLK had a meeting to discuss the themes identified and reached consensus. The finalized themes were then mapped to the broader themes partly based on TAM (Appendix 3):

- Perceived usefulness of online teaching during the COVID-19 pandemic
- Perceived ease of delivering online teaching during the pandemic
- Experience with institutional support for online teaching during the pandemic
- Acceptance of online teaching after the pandemic

## Findings and discussion

### Teachers’ experience with online teaching prior to the pandemic

As summarized in Table 3, approximately one-third of all survey respondents claimed to have never delivered online teaching prior to the COVID-19 pandemic (33.9% at HKUMed and 33.3% at UNSWMH). While this finding should not be interpreted as the rate of online teaching adoption in these institutes prior to the COVID-19 pandemic, it supports that there was a proportion of teachers who had never conducted online teaching prior to the COVID-19 pandemic, which is consistent with the DoI theory [13].

**Table 3:** Modalities of online teaching delivered by survey respondents before and during COVID-19 pandemic

	Frequency (Percentage)			
	HKUMed		UNSWMH	
	Before COVID-19	Since COVID-19	Before COVID-19	Since COVID-19
None	37 (33.9%)	N/A*	10 (33.3%)	N/A*
Lecture	57 (52.3%)	82 (75.2%)	13 (43.3%)	25 (83.3%)
Practical	11 (10.1%)	23 (21.1%)	6 (20.0%)	14 (46.7%)
Classroom tutorial	30 (27.5%)	74 (67.9%)	12 (40.0%)	24 (80.0%)
Bedside tutorial	4 (3.7%)	13 (11.9%)	0 (0%)	1 (3.3%)
Teaching in clinical areas	6 (5.5%)	14 (12.8%)	0 (0%)	3 (10.0%)
Others (e.g., physical examination skills demonstrations, fully online modules, webinars)	8 (7.3%)	8 (7.3%)	4 (13.3%)	4 (13.3%)
Total number of respondents	109		30	

^*^ Survey respondents who have not delivered online teaching during COVID-19 pandemic were excluded from the study

### Perceived usefulness of online teaching during the pandemic

Participants in our study delivered online teaching in multiple formats during the pandemic, ranging from online lectures, small-group tutorials, practical classes, and clinical teaching (Tables 2 and 3). Some survey respondents and interviewees indicated that online teaching enabled them to maintain some teaching activities despite the suspension of face-to-face classes. Most interviewees mentioned that they delivered their lectures as either synchronous online lectures or asynchronous videos. On the other hand, CT2 (“early adopter”), CT3 (“innovators”) and CT6 (“innovators”) described how they delivered some clinical skill training via online video-conferencing platform, while CT5 (“early adopter”) and NT7 (“innovator”) adapted some of their large-size practical classes into series of online small-group tutorials.

With regards to the benefits of the online teaching format, most interviewees felt asynchronous online lectures provided more flexibility to students, allowing them to pause or re-watch the videos anytime. This sentiment is consistent with other studies on medical students’ online learning experience during the COVID-19 pandemic [32, 33]. Furthermore, some interviewees described how synchronous online lectures enabled them to maintain some interactions with students. CT2 (“early adopter”) found that while some students tend to be less outspoken in face-to-face lectures, the synchronous online lecture format provided new ways for those students to interact with their teacher. Students were able to ask or answer questions non-verbally and/or anonymously. CT5 (“early adopter”) and NT7 (“innovator”) found their students preferred the online small-group tutorials to large-sized face-to-face classes, because there were more opportunities to interact with tutors/demonstrators. Interestingly, CT5 (“early adopter”) and NT6 (“laggard”) found that the attendance rate for some of their tutorials increased since switching to online teaching, and they presumed this was because students no longer had to commute to the campus.

However, several survey respondents and interviewees also described the difficulties and limitations of online teaching. There were 6 interviewees mentioning that a fully online format was not effective for practical skills teaching and experiential learning. Most interviewees who were clinical teachers mentioned that it was difficult to teach physical examination skills using an online setting, whereas a face-to-face setting would have allowed students to practice their physical examination skills on other students.

*“I think the main thing is the clinical skills transfer… for internal medicine where we're doing our respiratory exam, abdominal exam, it's better if you can see the patient, I mean, we can go through the exam with the students so that they can understand what they should be listening for, what they should be feeling for. And sometimes it's difficult to [bring] across that through videos and also just some online discussion.”*

- CT2, *interview transcript*

Furthermore, there were 5 interviewees who mentioned that in an online videoconference setting, they were not able to pick up non-verbal cues from students as much as in a face-to-face setting, especially when the students’ web cameras were turned off. Without non-verbal cues, they were unable to gauge students’ understanding and participation; and as a result, they could not adjust their teaching accordingly. In addition, 7 interviewees mentioned they felt it was more difficult to have spontaneous teacher-student and student–student dialogues in an online setting, especially for large classes.

*“With face-to-face, if I am giving instructions, and I see a lot of blank looks, or people are not sure… you can kind of adjust yourself or you can kind of calibrate the teaching as you go along based on the students. With Zoom is much more difficult sometimes… especially when they've got their video turned off. You can't kind of calibrate that as well.”*

- NT5, *interview transcript*

As argued by Venkatesh and Davis [34], perceived usefulness is influenced by a number of factors, such as voluntariness of use, job relevance, output quality and result demonstrability. In this study, while the adoption of online teaching was non-voluntary due to the pandemic, some interviewees found online teaching effective for their teaching (job relevance) based on signs of students’ engagement with online learning and positive feedback (output quality and result demonstrability). However, some other interviewees found online teaching ineffective, because they found it difficult to interact and engage with their students (output quality and result demonstrability). Sentiments about the lack of non-verbal communication in online teaching were noted in publications by other medical teachers [35]. Moreover, most participants expressed it was difficult to deliver hands-on skills and experiential learning completely through online platforms (job relevance). Teachers and students’ perception about the limitation of online teaching for practical skill transfer is well reported in the literature. Several studies reported that medical students felt online teaching was not effective in teaching clinical skills [32, 36, 37], and publications by medical teachers also showed similar sentiment [38, 39]. However, similar to the comments made by CT2, CT3 and CT6 as described earlier, there were also reports on innovative approaches to teach and assess clinical skills online [2, 26, 40–45]. Our results also showed that interviewees of all innovation adopter categories were cognizant of both the strengths and limitations of online teaching in the context of medical education.

### Perceived ease of delivering online teaching during the pandemic

Questions 7 and 8 of the survey (Appendix 1) examined teachers’ perceived ease of delivering online teaching during the pandemic. At both institutes there were more than 50% of survey respondents indicating that they felt either “very prepared” or “prepared” when they were asked to switch to online teaching in response to the COVID-19 outbreak (Table 4, Q7). Similarly, more than 75% of all survey respondents indicating that they felt technically “very equipped” or “equipped” to deliver online teaching (Table 4, Q8). As shown in Table 5, bivariate correlation between Q7 and Q8 showed a statistically significant positive correlation between Q7 and Q8 (*r*_s_ = 0.524, p < 0.01), suggesting a positive correlation between technical capability and preparedness for online teaching.

**Table 4:** Teachers’ experience with online teaching. Descriptive statistics

		Frequency (Percentage)		
		HKUMed	UNSWMH	Total
**Q7.** When asked to deliver online teaching, did you feel prepared?	Very prepared	6 (5.5%)	5 (16.7%)	11 (7.9%)
	Prepared	71 (65.1%)	12 (40.0%)	83 (59.7%)
	Unprepared	31 (28.4%)	7 (23.3%)	38 (27.3%)
	Very unprepared	1 (0.9%)	6 (20.0%)	7 (5.0%)
**Q8.** Did you feel technically equipped to deliver online teaching?	Very equipped	12 (11.0%)	6 (20.0%)	18 (12.9%)
	Equipped	71 (65.1%)	17 (56.7%)	88 (63.3%)
	Unequipped	25 (22.9%)	6 (20.0%)	31 (22.3%)
	Very unequipped	1 (0.9%)	1 (3.3%)	2 (1.4%)
**Q9.** How satisfied were you with the institutional support for e-learning during this class suspension?	Very satisfied	20 (18.4%)	6 (20.0%)	26 (18.7%)
	Satisfied	73 (67.0%)	18 (60.0%)	91 (65.5%)
	Dissatisfied	15 (13.8%)	6 (20.0%)	21 (15.1%)
	Very dissatisfied	1 (0.9%)	0 (0%)	1 (0.7%)
**Q10.** As a result of your experience, will you be more or less inclined to use e-learning in place of your former teaching modality after resumption of normal teaching arrangements?	Much more inclined	7 (6.4%)	6 (20.0%)	13 (9.4%)
	More inclined	46 (42.2%)	14 (46.7%)	60 (43.2%)
	Neither more nor less inclined	38 (34.9%)	8 (26.7%)	46 (33.1%)
	Less inclined	11 (10.1%)	2 (6.7%)	13 (9.4%)
	Much less inclined	7 (6.4%)	0 (0.0%)	7 (5.0%)
	Total number of respondents	109	30	139

Total number of respondents = 139

**Table 5:** Teachers’ experience with online teaching. Spearman’s rank correlation analysis

		**Q7**	**Q8**	**Q9**	**Q10**
**Q7**	Correlation (*r*_S_)	1.000	0.524**	0.176*	0.103
	p-value (2-tailed)	-	3.5571 × 10^–11^	0.038	0.226
**Q8**	Correlation (*r*_S_)	0.524**	1.000	0.305**	-0.004
	p-value (2-tailed)	3.557 × 10^–11^	-	0.000263	0.967
**Q9**	Correlation (*r*_S_)	0.176*	0.305**	1.000	0.064
	p-value (2-tailed)	0.038	0.000263	-	0.454
**Q10**	Correlation (*r*_S_)	0.103	-0.004	0.064	1.000
	p-value (2-tailed)	0.226	0.967	0.454	-

^**^ Significant correlation with *p* < 0.01

^*^ Significant correlation with *p* < 0.05

In the in-depth interviews, some interviewees stated that their online teaching ran quite smoothly. CT4, a “late majority” who had never delivered online tutorials before the pandemic, mentioned they were able to adapt to running online tutorials on Microsoft Teams quite quickly. CT3, an “innovator” who pioneered online clinical teaching at their institute, while initially concerned the technical issues may compromise students’ experience, found that online clinical teaching actually enabled students to successfully practice history taking with volunteer patients.

When asked to describe the challenges they faced with online teaching, 5 interviewees discussed about the hardware and infrastructure limitations they faced in their online teaching. The key issues identified include inadequate devices, limited internet bandwidth and lack of access to quiet space. NT1 (“late majority”) said it was difficult to find a quiet place outside the university campus. Whilst delivering a synchronous online lecture at home, NT1 experienced distractions from family members and neighbors, as well as the background noise on their students’ end. NT4 (“early majority”) mentioned that they only had access to limited equipment and a shared office in the campus, which were inadequate for their online teaching practice. As a result, NT4 resorted to recording online lectures and conducting online tutorials at home using their own devices. NT4 also mentioned their online teaching was sometimes interrupted by the limited internet bandwidth at home. When asked to suggest ways to improve teachers’ online teaching experience, NT4 suggested that universities should provide dedicated quiet space for lecture video recording and conducting online teaching, while CT2 suggested that universities should provide audiovisual equipment loaning service for teachers who do not already have their own equipment.

In addition, 9 interviewees mentioned that they found online teaching itself and the preparation of online teaching resources very time-consuming and required additional effort. NT1 (“late majority”) found recording a video lecture more difficult than delivering a face-to-face lecture, because it was easy to make mistakes while recording a video lecture, which would lead to multiple rounds of re-recording. NT7 (“innovator”) also shared similar sentiment, and they also felt that there is a lower tolerance for mistakes in pre-recorded lecture video compared to a synchronous or face-to-face lecture.

*“[People] are very conscious of the fact that it's being recorded. So, you know, a face-to-face is a little bit more forgiving, you say, ‘Oh sorry, I've got that wrong!’ you know, this is what it is; whereas rather than including all of those things in a pre-recorded lecture, people might say, ‘oh, let's go back and do that again.’… people often tend to go back and restart, redo it again… So yeah, people tend to prepare differently, if they know it's going to be recorded, compared to just turning up and delivering the face-to-face lecture…”*

- NT7, *interview transcript*

Overall, our findings suggested that teachers’ perceived ease of delivering online teaching depends on multiple internal and external factors. We found that individual teachers’ technical capability was significantly correlated with their sense of preparedness to deliver online teaching. This was expected because according to the DoI theory, individuals who are more comfortable with technology tend to adopt innovation or new technology sooner [10, 11, 13]. On the other hand, interviewees of various innovation adopter categories generally found online teaching and the preparation of online learning resources demand more time and effort, in comparison to the traditional face-to-face-only format. This sentiment is echoed by literatures about teachers’ opinions on online teaching [46, 47], and some argued that developing a course with blended learning would take 2 to 3 times longer than a similar course in face-to-face-only format [48, 49]. Some interviewees also mentioned that limited access to equipment and infrastructure was a key challenge in online teaching during the pandemic, and a small number of interviewees resorted to acquiring their own personal equipment to deliver online teaching at home. Overall, our findings are consistent with literatures on the drivers and barriers to the adoption of online teaching in institutes, where time constraint, poor technological literacy, inadequate infrastructure, and limited equipment availability were identified as barriers to adopting online teaching [9, 10, 14–17].

### Institutional support

When asked to indicate their level of satisfaction with institutional support for online teaching, more than 80% of survey respondents indicated that they were “very satisfied” or “satisfied” with the institutional support during the pandemic (Table 4, Q9). In addition, as shown in Table 5, bivariate correlation indicates a statistically significant positive correlation between a teacher’s perceived technical capability (Q8) and their level of satisfaction with institutional support (Q9), *r*_s_ = 0.305, p < 0.01. This finding suggests that teachers who felt comfortable with technology were more likely to be satisfied with the institutional support they received; whereas the teachers who felt less comfortable with technology were less likely to find it satisfactory.

In the in-depth interviews, NT6 (“laggard”), who explicitly claimed to be uncomfortable with technology, mentioned that they could not learn much from the online training sessions offered by their institute, and they only managed to become proficient at conducting online tutorials after several one-on-one training sessions. NT7 (“innovator”) also observed a similar trend among their colleagues.

*“The biggest challenge a lot of people had was feeling really stressed and very anxious, because they just didn't have the experience with their technology. A lot of people caught up quite quickly, but I think a lot of people still are struggling and are still working towards getting that… It's not enough to rely on people taking up just the training opportunities, because often those training opportunities just not at a time when they need it.”*

- NT7, *interview transcript*

In contrast, several interviewees mentioned they had to self-learn how to teach online independently. NT2 (“late majority”) described their process of learning how to teach online as a “sink-or-swim situation”. CT3 (“innovator”) also described similar situation in when converting their clinical teaching to online delivery.

*“I pretty much started this [online clinical teaching] on my own, so there's no direction and initiative. There's no additional support. I basically arranged everything more or less single-handedly. And then, on the way we learned and we adapted and we asked for additional software and hardware support from the department. So, I mean, in those times early on in the pandemic, everything was a mess. And there were colleagues who were hoping this would be a transient phenomenon and we can probably catch up with the schedule once school resumes. But at that time, of course, we never... never completely knew or believed that it would drag on forever. So that took a while to become a pressing need.”*

- CT3, *interview transcript*

Several interviewees who were comfortable with technology also found that the information technology (IT) support staff in their institute could not advise them on how to apply different technology or software features in their teaching practice. CT3, a clinical academic who showed “innovator” traits, expressed that even though the IT team had some level of understanding about the hardware and software, they were not familiar with how each function can be applied in different pedagogical scenarios, especially for medical and healthcare education.

*“Even if the IT teams are there, they are not familiar with the functions and how it actually works out during different teaching and learning scenarios. […] So for example, the annotate function or the remote control function, I didn't know it was there until say two or three weeks after I started the sessions. So at the first two sessions I didn't have these functions to use. But then I read and I found out all these adjunct functions on Zoom platform that's available, and I incorporated them to the teaching and found them particularly useful. So that is something that the tech support staff may not be able to help me with.”*

- CT3, *interview transcript*

Furthermore, NT7 (“innovator”) found it more helpful to discuss online teaching and educational technology with peers of their own disciplines, and they suggested that teachers who are more experienced with educational technology could mentor those who are less so. This sentiment is consistent with evidence in the literature, as it was reported that the community of practice model had significantly better impact in promoting the use of technology in teaching compared to the workshop model [50, 51].

*“I think that having someone in your discipline is always more valuable because they speak the same language. And, you know, you can actually talk about solving the problem rather than trying to explain what you're trying to do first.”*

- NT7, *interview transcript*

Overall, our findings suggest that individuals of different innovator categories or different technological literacy have different requirements of institutional support. “Late majorities”, “laggards” or teachers with low technological literacy generally need just-in-time IT support to get familiarized with the basics of online teaching (e.g. web conferencing), and this is consistent with another study arguing that just-in-time technical support is crucial for teachers with lower technological literacy [52]. Whereas for “innovators” who are exploring innovative ways of doing online teaching, IT support alone may not fully address their needs.

### Acceptance of online teaching after the COVID-19 pandemic

Question 10 of the survey (Appendix 1) looked at teachers’ inclination to adopt online teaching after the pandemic. As shown in Table 4, more than 50% of all survey respondents indicated that they felt “much more inclined” or “more inclined” to adopt online teaching after the resumption of normal teaching arrangements, while 14.4% indicated they felt “much less inclined” or “less inclined”. Bivariate correlation analysis showed no statistically significant correlation between question 10 and the other questions, indicating that in this study, teachers’ inclination to adopt online teaching after the pandemic was not correlated with their level of satisfaction with institutional support during the pandemic (predominantly IT support, self-access resources or training workshops) and their perceived technical capability to deliver online teaching (Tables 4 and 5). While TAM depicts a causal relationship between perceived ease of use and acceptance of technology [18, 19], the quantitative results do not substantiate such a relationship in this study.

When we asked the interviewees to describe their perspectives on possible teaching practices after the COVID-19 pandemic, many of them indicated their openness to keep delivering online teaching to various degrees, while resuming face-to-face delivery for some activities. CT4 (“late majority”) mentioned while they enjoyed face-to-face interactions in class, having experienced online teaching during the pandemic, they felt online delivery could be useful for mitigating the geographical barriers for some of their students who live remotely. CT1 (“early majority”), CT4 (“late majority”) and CT5 (“early adopter”) explicitly mentioned they plan to make their lectures asynchronous, while having some online interactive classes to complement their asynchronous lectures.

*“And now that we've converted all our lectures virtually to an online format... instead of having like an hour-long or two-hour-long lecture, we've got it in smaller bites, which is easier for the students to just do in sections - that will stay... and I suppose COVID helped to keep that. But I think the university wanted that anyway. But what we may still do is keep this sort of online student support sessions, which would have been like an end of a lecture or during a lecture where students can ask questions, they can then still ask those questions and be involved in that. And whether we do the tutorials still online, it may be that we do some online, some face-to-face for the students that want to physically come in. But yeah, the practicals still get them face-to-face.”*

- CT5, *interview transcript*

All clinical teachers generally agreed that it is imperative for physical examination skill training and bedside teaching to be delivered face-to-face. However, CT1 (“early majority”) and CT3 (“innovator”) perceived that online or digital technology could potentially enhance their face-to-face clinical teaching. CT1 and CT3 described that in bedside teaching before the pandemic, it was difficult to display a patient’s medical history and/or test results to their students, because there is only one hard copy of these materials but many students in the ward. They felt using digital devices and platforms can mitigate this problem. CT3 and CT6 (“innovators”) also perceived the usefulness for some clinical teaching to be delivered fully online even after the pandemic, because the COVID-19 pandemic has increased the popularity of teleconsultation in day-to-day clinical practice. Such changes would drive the need to include teleconsultations skills in clinical curricula, which in turn will require medical and healthcare students to practice online consultation techniques.

*“I have a colleague in primary care… his remark in March [2020] was we've achieved in 10 weeks what would have taken more than 10 years, in terms of the uptake of telehealth… Everybody just flipped - they literally went from having a patient across the desk to having a patient on the screen, and it was essentially overnight.”*

- CT6, *interview transcript*

However, some teachers remained resistant to online teaching. NT2 (“late majority”) expressed openness to keeping lectures as asynchronous online videos, but they were cautious of delivering small-group tutorials online in non-emergency situations, because they thought their tutorials genuinely require seamless face-to-face discussion, and this was confounded in the online format during the pandemic. NT6 (“laggard”) felt that online teaching during the pandemic was a necessary evil, and they were skeptical about having a substantial amount of teaching delivered online in non-emergency situations.

*“I would be very careful of moves to put a substantial amount of teaching online. I think the lesson I got from it is, it can be done, but it's not as good. You know, we had to do it this year, but it's not as good. I think the students struggled with it. I think they are very isolated because of it. I think as I said, as colleagues… as staff we got very isolated because of it. So I think... any moves to put substantial amounts of the syllabus online are not good. Small amounts, yes. But I think that the majority of our teaching needs to stay face-to-face. There's too much value in it to throw it out.”*

- NT6, *interview transcript*

Overall, based on the survey and in-depth interview data, we observed various levels of acceptance towards online teaching after the pandemic. Several interviewees (ranging from “innovator” to “late majority”) felt that online deliver was effective for some of their teaching. Interviewees in the “innovator” and “early adopter” categories also described how they would build on their online teaching experience in the pandemic to implement blended learning in their own context. Two interviewees (“late majority” and “laggard”) expressed resistance towards online teaching, because of their belief that online teaching is inferior to face-to-face teaching. In contrast, CT3 and CT6 (“innovators”) argued that since the pandemic has transformed day-to-day medical practice, medical and healthcare students will need to develop online consultation skills, therefore some synchronous online teaching is still necessary; and this sentiment is also shared by other medical and healthcare education institutes [53–55].

While an individual’s acceptance of technology is influenced by perceived usefulness and perceived ease of use according to TAM [18, 19], our findings did not demonstrate a causal relationship between perceived ease of delivering online teaching and acceptance of online teaching after the pandemic. Instead, our findings suggest that individual teachers’ acceptance of online teaching in after the pandemic is influenced by perceived usefulness of online teaching. On the other hand, a community of practice could allow teachers who are more experienced in online teaching to share their experience with those who are less so, and this may potentially shape the teacher community’s perceived usefulness of online teaching and intention to adopt online teaching; as social influence has been shown to influence individuals’ perceived usefulness and intention to adopt a technology [56].

### Strengths and limitations

The strength of this study is the sequential mixed method design with online survey followed by individual in-depth interviews. The qualitative component of this study allowed us to learn about individual teachers’ experience and perspectives in great depth, which is not afforded in online surveys collecting both quantitative data and open-ended responses. However, a major limitation of this study was the low approximate response rate of the online survey and small sample size for in-depth interviews. Also, all participants took part in this study on a voluntary basis, so that the voluntary response bias also prevented us from having a representative picture of teachers’ experience with online teaching during the pandemic and their acceptance of online teaching after the pandemic at both institutes. Another limitation of this study was the lack of demographic details such as age and gender in the quantitative part of this study. For the qualitative part of this study, these details were not reported in this paper due to re-identification risk.

## Conclusion

This paper evaluated medical and healthcare teachers’ experience in emergency remote teaching during the COVID-19 pandemic and their attitude towards online teaching after the pandemic. Overall, while the teachers at HKUMed and UNSWMH were adapting to online teaching, some had to learn the basics of online teaching, while others managed to develop innovative ways of online teaching. Teachers who participated in this study generally found it difficult to teach physical examination and practical skills entirely online. Interaction with students, lack of equipment, infrastructure and support, addition time and effort required for preparation were some of the key difficulties faced by teachers when delivering emergency remote teaching. Different teachers also have different needs for support based on their technological literacy, highlighting the need for institutes to be mindful of the diverse needs among different teachers. Our findings also suggest that teachers’ acceptance of online teaching in non-emergency situation after the pandemic was likely to be influenced by perceived usefulness of online teaching. While this study focused on teachers’ experience, students’ online learning experience remains an important element in understanding the impact of the COVID-19 pandemic on medical and health sciences education worldwide. Therefore, further studies on students’ experience during the pandemic are warranted.

## Supplementary Information

**Additional file 1: Appendix 1.** Survey.

**Additional file 2: Appendix 2.** Interview schedule.

**Additional file 3: Appendix 3.** Themes identified in the in-depth interviews.

## Data Availability

The datasets generated and/or analyzed during the current study are not publicly available due to ethics limitations but are available from the corresponding author on reasonable request.

## Abbreviations

COVID-19: Coronavirus disease 2019
DoI theory: Diffusion of Innovations theory
HKU: The University of Hong Kong
HKUMed: Li Ka Shing Faculty of Medicine, The University of Hong Kong
IT: Information technology
TAM: Technology acceptance model
UNSW: The University of New South Wales
UNSWMH: Faculty of Medicine and Health, The University of New South Wales
