# Supplementary material for: Medical teachers’ experience of emergency remote teaching during the COVID-19 pandemic: a cross-institutional study
Source: BMC Med Educ. 2022 Apr 21;22:303. doi: 10.1186/s12909-022-03367-x (PMC9021818; doi:10.1186/s12909-022-03367-x)
Supplement: Supplementary file 1 — Additional file 1: Appendix 1. Survey. [file 12909_2022_3367_MOESM1_ESM.pdf]

Appendix 1. Survey

Q1. Are you a clinical or non-clinical academic?

- Clinical
- Non-clinical

Q2. Have you delivered e-learning activities prior to the COVID-19 pandemic?

- Yes (Proceed to question 3)
- No (Proceed to question 4)

Q3. How satisfied were you with the institutional support for e-learning?

- Very dissatisfied
- Dissatisfied
- Satisfied
- Very satisfied

Q4. Did the suspension of face-to-face teaching due to the COVID-19 pandemic change your plans to engage in e-learning delivery?

- Yes
- No

Q5. What teaching activities have you delivered using e-learning tools PRIOR to the suspension of face-to-face teaching from COVID-19 outbreak? Please check the relevant box(es) as necessary.

- None
- Lecture
- Practical
- Classroom tutorial
- Bedside tutorials
- Teaching in clinical areas
- Others: ______________

Q6. What teaching activities have you delivered using e-learning tools DURING the suspension of face-to-face teaching from COVID-19 outbreak? Please check the relevant box(es) as necessary.

- None
- Lecture
- Practical
- Classroom tutorial
- Bedside tutorials
- Teaching in clinical areas
- Others: ______________

(If the respondent answered "none" for question 6, this would be the end of the survey for them.)

Q7. When asked to deliver online teaching, did you feel prepared?

- Very prepared
- Prepared
- Unprepared
- Very unprepared

Q8. Did you feel technically equipped to deliver online teaching?

- Very equipped
- Equipped
- Unequipped
- Very unequipped

Q9. How satisfied were you with the institutional support for e-learning during the transition to online teaching during the COVID-19 pandemic?

- Very satisfied
- Satisfied
- Dissatisfied
- Very dissatisfied

Q10. As a result of your experience, will you be more or less inclined to use e-learning in place of your former teaching modality after resumption of normal teaching arrangements?

- Much more inclined
- More inclined
- Neither more nor less inclined
- Less inclined
- Much less inclined

Q11. Please comment regarding your e-learning experience.
