# Supplementary material for: Medical teachers’ experience of emergency remote teaching during the COVID-19 pandemic: a cross-institutional study
Source: BMC Med Educ. 2022 Apr 21;22:303. doi: 10.1186/s12909-022-03367-x (PMC9021818; doi:10.1186/s12909-022-03367-x)
Supplement: Supplementary file 2 — Additional file 2: Appendix 2. Interview schedule. [file 12909_2022_3367_MOESM2_ESM.pdf]

Appendix 2. Interview schedule

1. What are the teaching activities you are involved in at the moment?
2. How did you teach online during COVID-19 pandemic?
3. Was there one particular time you felt it went well?
4. What are the challenges you faced?
5. What are something about face-to-face teaching that online teaching cannot replace?
6. What are some of the lessons learnt during this period?
7. What do you think are some of the key skills for teachers nowadays?
8. Moving back to a normal scenario, how do you think we can make the most out of face-to-face and online teaching?
9. How do you think the university / faculty can support you better?
