# Supplementary material for: Medical teachers’ experience of emergency remote teaching during the COVID-19 pandemic: a cross-institutional study
Source: BMC Med Educ. 2022 Apr 21;22:303. doi: 10.1186/s12909-022-03367-x (PMC9021818; doi:10.1186/s12909-022-03367-x)
Supplement: Supplementary file 3 — Additional file 3: Appendix 3. Themes identified in the in-depth interviews. [file 12909_2022_3367_MOESM3_ESM.pdf]

Appendix 3. Themes identified in the in-depth interviews

**A1. Perceived usefulness of online teaching during the COVID-19 pandemic (positives)**	
**Themes**	**Excerpts**
Able to give more immediate feedback to students compared to large face-to-face classes	"What the students also said that they liked is polls […] we're getting them to answer questions, putting feedback in […] using the chat function, answering questions that way, so that they feel involved."
Effective for didactic teaching	"And if they watch them and they cannot understand, then they can re-watch them."
Effective for small group teaching	"I think it works quite well for very small groups where you can see individuals […] it's a much more natural dialogue."
Enhanced collaborative work between students	"We made extensive use of breakout groups. So we could have small group work with the students, and then they'd come back and report back to the large group."
More convenient and flexible	"You can listen to the lecture anytime you want, so you can choose when you can take the class."
Online teaching was better than expected	"Students provided a lot of really positive feedback […] they felt that they were able to engage with the material and ask questions a lot more easily than they would have otherwise […] experienced previously with being in very large face-to-face classes."
Students became more engaged during face-to-face practical sessions	"So I think the students have been better behaved, and they've been more focused on the learning when they have come into the labs, because that's the only time they're coming in."
Students were more engaged during online lessons	"A lot of our students aren't willing to speak up [in a face-to-face lecture]. […] If we make it online, they can ask questions anonymously, and it allows me to tell everyone the question without naming the student. And so we can have a better discussion."

A2. Perceived usefulness of online teaching during the COVID-19 pandemic (negatives)	
**Themes**	**Excerpts**
Felt unsure about the quality of online teaching	"And I'm not sure how satisfactory the students may have found it. I did encourage them to give me feedback, but there wasn't any."
Difficult to engage with larger groups of students	"If you're in a live face-to-face tutorial […] just the fact that you go and talk to them and see them, you have eye contact, and you can see that they're either messing around, or they're working really hard, or they're looking confused, […] But you don't get that happening [in online tutorials]."
Online teaching was a compromise	"It has been challenging because [my discipline] […] really and truly requires there to be interactive dialogue […] for individuals to articulate their own views and thoughts and formulate their own ideas. And that actually is quite a difficult teaching modality to transfer to online."
Requires adjustment when converting face-to-face teaching to online teaching	"When I stand up in front of a group of students in a lecture, I can be far more animated. […] I can fill the room with activity, both my activity in front of them, but also engaging them and getting them to do activities. […] And that's something that you don't have when you're doing online. So you got to think of completely different ways of engaging the students online."
Sense of community lost without physical learning environment	"But I think the main problem is the interaction. You just don't get that face-to-face interaction. You don't get to know people. There's just a black circle on the screen with some initials and occasionally you see a picture of that person. And I found that very limiting."
Students did not turn on their microphone or web cameras during online lessons	"Whereas online, especially if they don't have their videos turned on, it's very difficult to understand whether you're getting through to them."
Students were disengaged during online lessons	"I think that was especially apparent in the initial stage where maybe it wasn't clear that this would be a long-term strategy. So I don't think a lot of the students took it as a serious learning experience."
Unable to teach hands-on practical skills:	
laboratory classes	"There's something about having your hand […] controlling the experimental stimulation and seeing the outcome immediately. With a video, you've just that one step removed."
physical examination skills / bedside teaching	"One important aspect of this clinical teaching […] is for them to learn and practice the actual physical examination skills. So that part is extremely difficult to translate [to online teaching]."

B. Perceived ease of delivering online teaching during the pandemic	
**Themes**	**Excerpts**
Felt self-sufficient in online teaching	“For me, it is not... it is always not an issue, because we use [web conferencing] on a day-to-day basis.”
Limited access to support from other colleagues	“It's been very stressful. And I think because all the staff have been at home and not together, we haven't been able to support each other as much.”
Requires more time and effort than face-to-face teaching	“[Recording video lectures] is actually more difficult than delivering a real-time lecture. […] You will be making a lot of mistakes in your speech. […] You'll go back and record it again, again and again. […] You spend the whole afternoon doing that 1-hour recording.”
Limitations of software features	“What I find weird about Teams is that I cannot see the participants at the same time as I'm sharing my screen. So if I'm sharing my screen to present a PowerPoint, I can't find how to keep track of who's listening online.”
Unfamiliar with the technology	“when we were first told we had to move our tutorials online, I had no idea how we were going to do that. Because IT is not my expertise, but I did get help. I think I took about four lessons to learn how to use Blackboard Collaborate, and eventually somebody got hold of me and taught me one-to-one and that worked. The other lessons were... I don't have a clue what's going on. And that's partly because likely, no, I'm not great with IT skills.”

C. Experience with institutional support for online teaching during the pandemic	
**Themes**	**Excerpts**
IT support not meeting their needs	"Even if the IT teams are there, they are not familiar with the functions and how it actually works out during different teaching and learning scenarios. […] the teacher really need to know the abilities of the platform, and how to use those different functions to replace or add onto the actual teaching delivery."
Internet connectivity problem	"[Internet connectivity] is not just the teachers' problem. Students' connections - you can't control. […] you can't make sure that they have good connections"
Lack of devices / equipment	"So at home, I have two monitors, so I can say, okay, one monitor is just looking at other people on Zoom, and the other have the documents of PBL case opened, then I can do that. Whereas if I have only one monitor, whenever, then that's pretty difficult."
Lack of quiet space	"First of all, you could have kids at home. You could have distractions at home, that suddenly someone yells from behind. You can't really have a lecture like that, you know. That's very unprofessional."
Peer collaboration / support from other teachers is important	"I also suggest to people that are just starting out, 'don't try and do it all yourself.' It's overwhelming. You are going to be far better off working in a team of people, people that have got more experience. […] having a group of people around you that allows you to share understanding, ask questions, and you know, just building your expertise along the way. It's going to be a whole lot easier if you've got a team of people around you."
Teachers had to self-learn how to teach online	"It was a bit sink or swim with some of the technology. Certainly things like Zoom, obviously, were new to me. […] I have felt I've had to navigate that reasonably independently. […] I guess I'm perhaps not as aware where I would access support for those sorts of things."

D. Adoption of online teaching after the pandemic	
**Themes**	**Excerpts**
Increased technology use in clinical practice (telemedicine) will necessitate more online teaching	“I think even if we move completely back to face-to-face, as if COVID never existed […] I think telemedicine is something that still needs to stay, and we have to teach students how to be master of this real sort of consultation skills.”
Online teaching requires deliberate planning	“Video recording of maybe core materials, like just maybe factual things, or explanation kind of things. And then after the students have learned that, then we move on to more discussion and application side. I think that for application side and that, face-to-face will be better.”
Open to adopting more online teaching	“I think what we might see is more of a shift towards a flipped classroom. […] I think the fact that people now have had to deliver things online, they've got resources that they could use in a way that will allow for flipped classroom delivery, in a way that they previously didn't have.”
Skeptical about adopting more online teaching	“I would be very careful of moves to put a substantial amount of teaching online. I think the lesson I got from [the pandemic] is, it can be done, but it's not as good. […] I think the students struggled with it. I think they are very isolated because of it. […] I think that the majority of our teaching needs to stay face-to-face. There's too much value in it to throw it out.”
Teachers need to become proficient in online teaching	“I think certainly, you need be technology savvy. You need to be quite creative in the way you deliver teaching. Perhaps more so than before, because it can't just be, as you describe, a didactic lecture anymore... there has to be more... perhaps thinking around you know, how you engage students with using the different modalities.”
